# Protection of flunarizine on cerebral mitochondria injury induced by cortical spreading depression under hypoxic conditions

**DOI:** 10.1007/s10194-011-0300-1

**Published:** 2011-02-25

**Authors:** Fengpeng Li, Enchao Qiu, Zhao Dong, Ruozhuo Liu, Shiwen Wu, Shengyuan Yu

**Affiliations:** Department of Neurology, Chinese PLA General Hospital, Beijing, 100853 China

**Keywords:** Cortical spreading depression, Mitochondria, Flunarizine, Hypoxia

## Abstract

A rat cortical spreading depression (CSD) model was established to explore whether cerebral mitochondria injury was induced by CSD under both normoxic and hypoxic conditions and whether flunarizine had a protective effect on cerebral mitochondria. SD rats, which were divided into seven groups, received treatment as follows: no intervention (control Group I); 1 M NaCl injections (Group II); 1 M KCl injections (Group III); intraperitoneal flunarizine (3 mg/kg) 30 min before KCl injections (Group IV); 14% O_2_ inhalation before NaCl injections (Group V); 14% O_2_ inhalation followed by KCl injections (Group VI); 14% O_2_ inhalation and intraperitoneal flunarizine followed by KCl injections (Group VII). Following treatment, brains were removed for the analysis of mitochondria transmembrane potential (MMP) and oxidative respiratory function after recording the number, amplitude and duration of CSD. The duration of CSD was significantly longer in Group VI than that in Group III. The number and duration of CSD in Group VII was significantly lower than that in Group VI. MMP in Group VI was significantly lower than that in Group III, and MMP in Group VII was significantly higher than that in Group VI. State 4 respiration in Group VI was significantly higher than that in Group III, and state 3 respiration in Group VII was significantly higher than that in Group VI. Respiration control of rate in Group VII was also significantly higher than that in Group VI. Thus, we concluded that aggravated cerebral mitochondria injury might be attributed to CSD under hypoxic conditions. Flunarizine can alleviate such cerebral mitochondria injury under both normoxic and hypoxic conditions.

## Introduction

Cortical spreading depression (CSD) is a self-propagating wave of cellular depolarization that has been implicated in migraine and in progressive neuronal injury after stroke and head trauma.CSD which was first described by Leao [1], comprises a wave of reversible EEG suppression that propagates at a rate of 2–3 mm/min across the cortical surface, accompanied by a negative deflection of the direct current (DC) potential and a severe disruption in ion homeostasis [2, 3]. Recovery from CSD depends upon ion pump activity to cause an increase in metabolic activity and oxygen demand [4–6]. In normal brain tissue, this chain of events is partially compensated by an increase in cerebral blood supply [6, 7]. This coupling will be disturbed in tissues which lack oxygen. Mitochondria are key to both apoptotic and necrotic brain cell death. Several prominent mitochondrial alterations, including changes in mitochondrial respiratory function and mitochondrial membrane potential (MMP) [8], accompanied by hypoxia can contribute to cell death. A variety of electrophysiological, visual and psychological events have indicated that CSD-like events occur during the initiation of migraines [9–13], and usually, but not always, a cerebral oligemia is the only cerebral vascular response consistently detected in these circumstances [14–16]. Cellular hypoxia, in turn, can cause an increase in the flow of calcium from the extracellular fluid to the intracellular space, resulting in calcium overload and cellular dysfunction. The piperazine derivative flunarizine, a non-selective calcium antagonist [17], shown in many controlled clinical studies to be effective in migraine prophylaxis [18–26], has been found to protect brain tissue against hypoxic damage [27]. Therefore, the purpose of this study is to explore whether cerebral mitochondrial injury is related to CSD under both normoxic and hypoxic conditions and to find whether flunarizine has a protective effect on cerebral mitochondria.

## Methods

This study on animals complies with the prevailing standards of animal welfare embodied in UK laws governing animal experimentation.

### Rats’ surgical preparation

CSD male rats (220–250 g) were placed in individual cages in a thermoregulated (22°C), 12 h night/12 h day room. They were allowed free access to water but fasted for 12–16 h before surgery. Rats (*n* = 58) were anesthetized with urethane via a 20% oxygen-balance nitrogen mixture. Then they were warmed by a heating lamp (37 ± 0.5°C) above their dorsal surface during surgical procedures and controlled via a rectal temperature probe. A tail artery was cannulated for sampling blood and monitoring arterial blood pressure.

A 2–3 mm left parasagittal skin incision was made from the midline. The wound edges were spread and the skull bone scraped. Then two small (1–2 mm diameter) craniotomies were drilled. The former was 2 mm lateral and 3 mm anterior to bregma, while the latter was 6 mm posterior and 5 mm lateral to bregma [28]. Both craniotomies were carefully drilled so that the underlying dura would not be torn. CSD was induced by microinjection of KCl via a microinjector placed 1 mm below the pial surface of the posterior craniotomy. 0.5 μl KCl (1 M) was injected every 10 min within 1 h. Rats of Group II or Group V were injected with NaCl (1 M) at an analogous dose and frequency. A glass micropipette (tip diameter, 1 μm; filled with 0.5 M sodium acetate) was positioned 1 mm below the pial surface through the anterior craniotomy to monitor DC neocortical potentials and to confirm whether CSD occurred. Microelectrodes were connected to an amplifier system, and then DC signals were displayed on a chart recorder. An indifferent electrode was positioned in the left neck muscle.

### Animal groups

Fifty-six SD rats were divided into seven groups randomly with each group consisting of eight rats. Group I was the control group. Group II received 1 M NaCl injections to the left parietal cortex. Group III received 1 M KCl injections to the left parietal cortex. Group IV received an intraperitoneal dose of flunarizine (3 mg/kg) 30 min before KCl injections mentioned above in Group III. Group V inhaled 14% O_2_ for 30 min before 1 M NaCl injections. Group VI inhaled 14% O_2_ for 30 min before 1 M KCl injections. Group VII inhaled 14% O_2_ for 30 min and received an intraperitoneal dose of flunarizine (3 mg/kg) 30 min before the 1 M KCl injections.

### Mitochondrial oxidative respiratory function and MMP

Fresh mitochondria were isolated from cerebral cortex as described by Clark [29]. The brain was placed in an ice-cold isolation media containing 0.25 M sucrose, 0.5 mM EDTA (dipotassium salt) and Tris–HCl (pH 7.4) immediately after the removal. The cerebral cortex was finely minced with scissors and homogenized with isolation buffer in an all-glass Teflon homogenizer. The homogenate was centrifuged at 2,000×*g* for 3 min, and the supernatant was carefully decanted and saved. The pellet was resuspended in half of the original volume of isolation buffer and centrifuged in the way mentioned above. The supernatant was saved and the pellet was discarded. The pooled supernatants were centrifuged at 12,500×*g* for 8 min. The resulting pellet was resuspended in 3% Ficoll solution, layered in 6% Ficoll solution, and then centrifuged at 11,500×*g* for 30 min. The pellet was resuspended in an appropriate volume of isolation buffer, and centrifuged at 12,000×*g* for 8 min. All the procedures were completed at 0–4°C within 90 min. The concentration of mitochondrial protein was measured by the Lowry method. The purified mitochondrial fraction was used for mitochondrial respiration and mitochondrial membrane potential measurements.

Oxygen uptake of isolated mitochondria (nmol O_2_/min per mg protein) was determined by polarography using a Clark-type oxygen electrode at 28°C as described by Chávez [30]. The pellet of mitochondria obtained from final centrifugation was resuspended and transferred to the oxygen electrode chamber in 800 μl buffer which contained 100 mM KCl, 75 mM mannitol, 25 mM sucrose, 5 mM potassium phosphate, 0.05 EDTA (K^+^ salt) and 10 mM Tris–HCl (pH 7.4). 5 mM pyruvate plus 2.5 mM malate were included as substrates. Incubation conditions for determining state 3 (ST3) (ADP and substrates present) and state 4 respiration (ST4) (after ADP was depleted) were as defined by Chance and Williams [31]. Respiration control rate (RCR) is defined as ST3 divided by ST4.

Hundred microliter of mitochondria obtained from final centrifugation, 2.5 μM rotenone and 1 μM rhodamine 123 were resuspended and transferred to 1 ml of reaction media which contained 150 mM sucrose, 5 mM sodium succinate, 5 mM K_3_PO_4_, 20 mM HEPES and 5 mM MgCl_2_. 5 min later, the mixed solution was centrifuged at 5,000×*g* for 5 min at room temperature.

[Rhodamine 123]_out_ was made with a spectrophotometer at 500 nm. Membrane potentials (negative inside) were calculated by the Nernst equation: Mitochondria membrane potentials (MMP) = 59 log ([*X*]_in_/[*X*]_out_). [Rhodamine 123]_in_ was estimated from rhodamine 123 uptake assuming distribution into a matrix space of 1 μl/mg of protein [32].

### Statistical analysis

The data of duration of CSD in each group are expressed as median 25% quartile to 75% quartile, and statistical analysis is performed using K related samples test. The other data are expressed as means ± SEM, and statistical analysis is performed using ANOVA, followed by post hoc Fisher’s test. Software SPSS 13.0 is used. Significance is assumed at *P* < 0.05.

## Results

### Cortical spreading depression

**Fig. 1:**
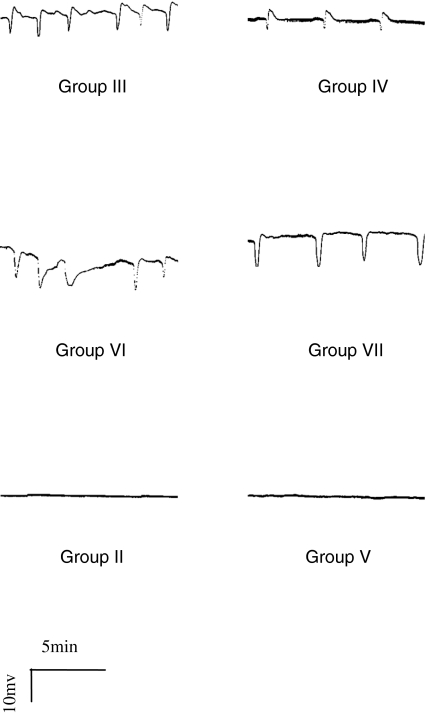
The manifestation of CSD

About 3 min after KCl injection, CSD was induced in rats of Group III, Group IV, Group VI and Group VII (see Fig. 1). The manifestation of CSD in Group III was an extracellular negative potential with amplitude of 10–30 mV and duration of 0.5–1.5 min, which may be preceded or succeeded by a positive deflection of variable amplitude and duration. The manifestation of CSD in Group IV, Group VI, and Group VII was similar to that in Group III. There was no CSD recorded in Group II and Group V.

The duration of CSD in Group V was significantly longer than that in Group III (*P* < 0.01). The number of CSD in Group IV was significantly less than that in Group III (*P* < 0.01). The amplitude of CSD in Group IV was significantly smaller than that in Group III (*P* < 0.05). The duration of CSD in Group IV was significantly shorter than that in Group III (*P* < 0.01). The number of CSD in Group VII was significantly less than that in Group VI (*P* < 0.05) (Table 1). The duration of CSD in Group VII was significantly shorter than that in Group VI (*P* < 0.01) (Table 2).

**Table 1:** The number and amplitude of CSD (mean ± SD)

Group	Number	Amplitude (mv)
Group III	9.25 ± 3.81	14.54 ± 6.05
Group IV	3.88 ± 4.05**	11.75 ± 4.13*
Group VI	8.63 ± 2.67	16.91 ± 6.12
Group VII	5.00 ± 3.16*^,Δ^	15.29 ± 3.39

* *P* < 0.05 versus Group III; ** *P* < 0.01 versus Group III; ^Δ^ *P* < 0.05 versus Group VI

**Table 2:** The duration of CSD in each group

Group	Duration (s)
Group III	59 (49.5–72.5)
Group IV	55 (50–62)*
Group VI	75 (64–92)**
Group VII	65 (58.5–79)**^,ΔΔ^

The values are median (25% quartile–75% quartile)

* *P* < 0.05 versus Group III; ** *P* < 0.01 versus Group III; ^ΔΔ^ *P* < 0.01 versus Group VI

### Mitochondrial oxidative respiratory function

ST3 in Group VI was significantly lower than that in Group I and Group II (*P* < 0.01). ST3 in Group VII was significantly higher than that in Group VI (*P* < 0.05). ST4 in Group VI was significantly higher than that in Group I and Group II (*P* < 0.01). RCR of Group VI was significantly lower than that in Group I (*P* < 0.01). RCR of Group VII was significantly higher than that in Group VI (*P* < 0.01) (Table 3).

**Table 3:** Mitochondrial oxidative respiratory function (mean ± SD)

Group	ST3 (nmol/min/mg pro)	ST4 (nmol/min/mg pro)	RCR
Group I	103.05 ± 21.73	18.29 ± 7.15	6.18 ± 2.02
Group II	102.27 ± 8.88	18.36 ± 5.66	6.04 ± 2.05
Group III	82.47 ± 24.00	29.26 ± 12.12	3.14 ± 1.46
Group IV	89.39 ± 33.95	29.16 ± 17.61	4.54 ± 1.06
Group V	93.35 ± 22.02	43.05 ± 16.68**^,ΔΔ^	3.30 ± 1.44
Group VI	61.73 ± 23.44**^,▲▲^	45.66 ± 18.11**^,^◊	1.36 ± 0.35**
Group VII	86.28 ± 19.28^☆^	37.62 ± 11.19**	2.34 ± 0.36*^,☆☆^

* *P* < 0.05 versus Group I; ** *P* < 0.01 versus Group I; ^ΔΔ^ *P* < 0.01 versus Group II; ^◊^ *P* < 0.05 versus Group III; ^▲▲^ *P* < 0.01 versus Group V; ^☆^ *P* < 0.05 versus Group VI; ^☆☆^ *P* < 0.01 versus Group VI

### MMP

MMP in Group III (*P* < 0.01) and Group VI (*P* < 0.05) was significantly lower than that in Group I. MMP in Group VI was significantly lower than that in Group III(*P* < 0.01). MMP in Group IV was significantly higher than that in Group III (*P* < 0.01). MMP in Group VII was significantly higher than that in Group VI (*P* < 0.01) (Table 4).

**Table 4:** MMP in each group (mean ± SD)

Group	MMP (mv)
Group I	194.30 ± 10.58
Group II	188.94 ± 7.25
Group III	161.89 ± 14.99**^,ΔΔ^
Group IV	179.06 ± 11.60*^,◊◊^
Group V	182.59 ± 8.21
Group VI	144.78 ± 19.20**^,◊◊,▲▲^
Group VII	174.27 ± 8.94**^,☆☆^

* *P* < 0.05 versus Group I; ** *P* < 0.01 versus Group I; ^ΔΔ^ *P* < 0.01 versus Group II; ^◊◊^ *P* < 0.01 versus Group III; ^▲▲^ *P* < 0.01 versus Group V; ^☆☆^ *P* < 0.01 versus Group VI

## Discussion

CSD is now recognized to be probably the neurophysiological substrate of classical migraine aura, and may be involved in migraines without a perceived aura as well, although it has also been a focus of intense investigation in relation to apparently diverse disease states including ischemic or hemorrhagic stroke and head trauma [33]. Several recent descriptions of CSD in humans in the setting of brain injury provide definitive evidence that this phenomenon can occur and have important functional consequence in the human brain. Mayevsky and colleagues [34] used an invasive multiparametric monitoring system to record reduction of electroencephalogram (EEG) amplitude, and an increase in extracellular K^+^ in a patient with severe head injury. Strong and colleagues [35] used electrodes placed near foci of damaged cortical tissue in patients with intracranial or subarachnoid hemorrhage to record propagated depression of electrocorticographic (ECoG) activity whose rate of spread was the same as that of CSD. Despite the longstanding assumption that CSD is the primary mechanism underlying the migraine aura, there has yet been no definitive demonstration of the electrophysiological features of CSD in a migraine patient. The spatial and temporal characteristics of migraine aura symptoms and of the propagated changes in blood flow and functional MRI signal observed in migraine patients are certainly consistent with CSD [36]. However, surface EEG recording in migraine patients, including a few documented patients with migraine with aura, have not demonstrated the classical electrophysiological features of CSD [37]. This may be because surface EEG does not have the sensitivity to detect CSD. Recent studies suggest that spreading depression could be the final common pathophysiological target for several established or investigational migraine prophylactic drugs [33]. Five clinically effective migraine prophylactic drugs (valproate, topiramate, propranolol, amitriptyline, methysergide) were shown to suppress SD susceptibility [38]. Flunarizine showed a somewhat dose-dependent CSD suppression [39].

The number of CSD represents the susceptibility of the cortex to CSD. In this study, the number of CSD under both normoxic and hypoxic conditions was examined. Decreasing the percentage of O_2_ from room air concentration (21%) to 14% did not change the number of CSD. Takano [6] observed that decreasing the percentage of O_2_ from room air concentration (21%) to 8% generated CSD without KCl injection. From the two experiments, we concluded that the susceptibility of cortex to CSD increased at 8–14% O_2_. The susceptibility of the cortex to CSD is also affected by many other factors including cytoarchitecture, maturity and distribution of transmitter systems, ion channels in the neuron membrane and pH [40].

The wave of CSD is produced by neuron dendritic persistent inward currents. The amplitude of CSD is regulated by glutamate-controlled channels, sodium channels and calcium channels [41, 42]. In the present study, the amplitude of CSD under normoxic and hypoxic conditions was examined. Decreasing the percentage of O_2_ from room air concentration (21%) to 14% did not change the amplitude of CSD. Therefore, we concluded that the amplitude of CSD was not affected by O_2_ concentration.

The duration of CSD is related to the repolarization of neurons and glial cells. During this study, we found an inverse correlation between O_2_ supply and the duration of CSD. The finding implied that O_2_ supply was the important factor for normalization of extracellular K^+^.

Flunarizine is reported to be the only calcium antagonist capable of protecting brain cells against hypoxic damage [42, 43]. Results of more than 10 open and almost 20 placebo-controlled trials of migraine prophylaxis with flunarizine demonstrate the beneficial effects and safety of this agent [18]. Meanwhile, eight clinical trials compared the calcium channel blocker with β-adrenoceptor blockers (six trials with propranolol, two with metoprolol) [19–26], in which flunarizine was equally effective with the β-adrenoceptor blockers, but had a qualitatively different adverse event profile. Hence, flunarizine is considered a drug of first choice in migraine prophylaxis, and is used as first-line prophylactic agents in most European countries [44].

In the present study, it was found that flunarizine decreased the number and amplitude of CSD, and shortened the duration of CSD under normoxic conditions. Also it was found that flunarizine decreased the number and duration of CSD under hypoxic conditions.

Flunarizine reduced the susceptibility of the cortex to CSD under normoxic and hypoxic conditions. Blockers of L-, N-, and P/Q-type voltage-gated calcium channels decreased the number of CSD [45]. These results suggested the effects of flunarizine on the number of CSD can be attributed to its blockers of L-, N-type voltage-gated calcium channels.

Flunarizine shortened the duration of CSD under both normoxic and hypoxic conditions, which may be attributed to its inhibitory effect on cortical hypoperfusion induced by CSD [46]. Thus, O_2_ supply in brain tissue is increased, which allowed the repolarization of neurons and glial cells.

Flunarizine decreased the amplitude of CSD under normoxic conditions, which may be attributed to the amplitude of CSD regulated by calcium antagonist [40]. Flunarizine did not decrease the amplitude of CSD under hypoxic conditions. This finding indicated that hypoxia attenuated the effect of calcium antagonists on the amplitude of CSD.

Whether CSD can cause irreversible neuronal injury or not is uncertain. As mentioned by Nedergaard and Hansen [47], in normally circulated and oxygenated healthy adult brains, SD can be provoked many times without obvious harm. Pomper et al. [48] report that repeated episodes of spreading depression-like events (SDLEs) caused irreversible injury in hippocampal slice cultures taken from immature brains. Mitochondria have many functions in eukaryotic cells. Perhaps the most important one is the generation of adenosine 5′-triphosphate (ATP) by oxidative phosphorylation. Since it had been shown that tissue ATP content decreases to 54% in rats during the passages of SD waves, the capability of tissue energy production has been studied [49]. The mitochondrial redox state inclines to the reduction side synchronous to the onset of CSD and normalizes within 120 s after the onset of depolarization by measuring the NADH fluorescence in vivo [50]. Along with many mitochondrial carrier-mediated processes, the generation of ATP requires an electrochemical potential generated by the electron transport chain. Thus, normally functioning mitochondria establish MMP and a pH gradient (ΔpH), which jointly comprises Δ*p*, the proton motive force that drives ATP synthesis. Processes that dissipate Δ*p* are usually considered harmful to cells. MMP is typically the more dynamic parameter in the proton motive force. MMP represents a common final pathway of many conditions associated with oxidative stress including hypoxia, hypoglycemia, and aging. MMP dissipation may be caused by ATP synthesis, Ca^2+^ transport, or the activity of other carrier proteins [51]. In this study, MMP dissipated due to CSD and further dissipated due to CSD under hypoxic conditions. Thus, our data indicated that CSD caused oxidative stress or aggravated hypoxic conditions in the brain. These changes can be attenuated by flunarizine.

ST3 reflects the maximum rate of coupled respiration, that is, when electron transport is coupled to ATP synthesis. ST4 reflects the rate of leakage of protons back across the inner mitochondrial membrane into the matrix. RCR reflects the coupling rate of oxidation phosphorylation. Our data indicated that CSD under hypoxic conditions decreased the maximal rate of coupled respiration, while increased the rate of leakage of protons back across the inner mitochondrial membrane into the matrix, and further uncoupled the mitochondria. The change of mitochondrial oxidative respiratory function by CSD under hypoxic conditions can partly be attenuated by flunarizine.

The exact pathogenesis of migraine is not determined and may be polymorphic. Platelet mitochondrial enzyme activities study has shown that NADH-dehydrogenase, citrate synthase and cytochrome *c* oxidase activities are significantly lower in migraineurs than in controls [52]. A deficient energy metabolism could be the missing link between the biobehavioral model and the hypoxia theory of migraine. It would explain why an excessive activation of cortical structures could lead to trigeminovascular activation via a metabolic disequilibrium, and possibly via hypoxia-sensing neurons and the hypoxia-inducible factor [53].

In conclusion, our work indicated that aggravated cerebral mitochondria injury might be induced by CSD under hypoxic conditions, and that flunarizine can alleviate cerebral mitochondria injury under both normoxic and hypoxic conditions. However, our work is limited to experimental animals and any link to migraine pathophysiology is purely hypothetical.

## References

[1] Leao AAP (1944). Spreading depression of activity in cerebral cortex. J Neurophysiol.

[2] Haghir H, Kovac S, Speckmann EJ, Zilles K, Gorji A (2009). Patterns of neurotransmitter receptor distributions following cortical spreading depression. Neuroscience.

[3] Chang JC, Shook LL, Biag J, Nguyen EN, Toga AW, Charles AC, Brennan KC (2010). Biphasic direct current shift, haemoglobin desaturation and neurovascular uncoupling in cortical spreading depression. Brain.

[4] Piilgaard H, Lauritzen M (2009). Persistent increase in oxygen consumption and impaired neurovascular coupling after spreading depression in rat neocortex. J Cereb Blod Flow Metab.

[5] Funke F, Kron M, Dutschmann M, Müller M (2009). Infant brain stem is prone to the generation of spreading depression during severe hypoxia. J Neurophysiol.

[6] Takano T, Tian GF, Peng W, Lou N, Lovatt D, Hansen AJ, Kasischke KA, Nedergaard M (2007). Cortical spreading depression causes and coincides with tissue hypoxia. Nat Neurosci.

[7] Sonn J, Mayevsky A (2000). Effects of brain oxygenation on metabolic, hemodynamic, ionic and electrical responses to spreading depression in the rat. Brain Res.

[8] Ouyang YB, Xu LJ, Sun YJ, Giffard RG (2006). Overexpression of inducible heat shock protein 70 and its mutants in astrocytes is associated with maintenance of mitochondrial physiology during glucose deprivation stress. Cell Stress Chaperones.

[9] Bartolini M, Baruffaldi R, Paolino I, Silvestrini M (2005). Cerebral blood flow changes in the different phases of migraine. Funct Neurol.

[10] Lauritzen M, Olsen TS, Lassen NA, Paulson OB (1983). Regulation of regional cerebral blood flow during and between migraine attacks. Ann Neurol.

[11] Lauritzen M (2001). Cortical spreading depression in migraine. Cephalalgia.

[12] Dalkara T, Zervas NT, Moskowitz MA (2006). From spreading depression to the trigeminovascular system. Neurol Sci.

[13] Mayevsky A, Doron A, Manor T, Mellin S, Zarchin N, Ouaknine GE (1996). Cortical spreading depression recorded from the human brain using a multiparametric monitoring system. Brain Res.

[14] Lauritzen M, Olsen TS, Lassen NA, Paulson OB (1983). Changes in regional cerebral blood flow during the course of classic migraine attacks. Ann Neurol.

[15] Olesen J, Larsen B, Lauritzen M (1981). Focal hyperemia followed by spreading oligemia and impaired activation of rCBF in classic migraine. Ann Neurol.

[16] Woods RP, Iacoboni M, Mazziotta JC (1994). Brief report: bilateral spreading cerebral hypoperfusion during spontaneous migraine headache. N Engl J Med.

[17] Gelmers HJ (1985). Calcium-channel blockers in the treatment of migraine. Am J Cardiol.

[18] Reveiz-Herault L, Cardona AF, Ospina EG, Carrillo P (2003). Effectiveness of flunarizine in the prophylaxis of migraine: a meta-analytical review of the literature. Rev Neurol.

[19] Grotemeyer KH, Schlake HP, Husstedt IW (1988). Prevention of migraine with metoprolol and flunarizine. A double-blind crossover study. Nervenarzt.

[20] Ludin HP (1989). Flunarizine and propanolol in the treatment of migraine. Headache.

[21] Shimell CJ, Fritz VU, Levien SL (1990). A comparative trial of flunarizine and propranolol in the prevention of migraine. S Afr Med J.

[22] Lutschg J, Vassella F (1990). The treatment of juvenile migraine using flunarizine or propranolol. Schweiz Med Wochenschr.

[23] Sørensen PS, Larsen BH, Rasmussen MJ, Kinge E, Iversen H, Alslev T, Nøhr P, Pedersen KK, Schrøder P, Lademann A (1991). Flunarizine versus metoprolol in migraine prophylaxis: a double-blind, randomized parallel group study of efficacy and tolerability. Headache.

[24] Gawel MJ, Kreeft J, Nelson RF, Simard D, Arnott WS (1992). Comparison of the efficacy and safety of flunarizine to propranolol in the prophylaxis of migraine. Can J Neurol Sci.

[25] Verspeelt J, Locht P, Amery WK (1996). Post-marketing cohort study comparing the safety and efficacy of flunarizine and propranolol in the prophylaxis of migraine. Cephalalgia.

[26] Bordini CA, Arruda MA, Ciciarelli MC, Speciali JG (1997). Propranolol vs flunarizine vs flunarizine plus propranolol in migraine without aura prophylaxis. A double-blind trial. Arq Neuropsiquiatr.

[27] Holmes B, Brogden RN, Heel RC, Speight TM, Avery GS (1984). Flunarizine a review of its pharmacodynamic and pharmacokinetic properties and therapeutic use. Drugs.

[28] Moskowitz MA, Nozaki K, Kraig RP (1993). Neocortical spreading depression provokes the expression of c-fos protein-like immunoreactivity within trigeminal nucleus caudalis via trigeminovascular mechanisms. J Neurosci.

[29] Clark JB, Nicklas WJ (1970). The metabolism of rat brain mitochondria. J Biol Chem.

[30] Chávez JZ, Pichiule P, Boero J, Arregui A (1995). Reduced mitochondrial respiration in mouse cerebral cortex during chronic hypoxia. Neurosci Lett.

[31] Chance B, Williams GR (1956). The respiratory chain and oxidative phosphorylation. Adv Enzymol.

[32] Emaus RK, Grunwald R, Lemasters JJ (1986). Rhodamine 123 as a probe of transmembrane potential in isolated rat-liver mitochondria: spectral and metabolic properties. Biochim Biophys Acta.

[33] Ayata C (2009). Spreading depression: from serendipity to targeted therapy in migraine prophylaxis. Cephalalgia.

[34] Mayevsky A, Doron A, Manor T, Meilin S, Zarchin N, Ouaknine GE (1996). Cortical spreading depression recorded from the human brain using a multiparametric monitoring system. Brain Res.

[35] Strong AJ, Fabricius M, Boutelle MG, Hibbins SJ, Hopwood SE, Jones R, Parkin MC, Lauritzen M (2002). Spreading and synchronous depressions of cortical activity in acutely injured human brain. Stroke.

[36] Charles A, Brennan K (2009). Cortical spreading depression-new insights and persistent questions. Cephalalgia.

[37] Lauritzen M, Trojaborg W, Olesen J (1981). EEG during attacks of common and classical migraine. Cephalalgia.

[38] Ayata C, Jin H, Kudo C, Dalkara T, Moskowitz MA (2006). Suppression of cortical spreading depression in migraine prophylaxis. Ann Neurol.

[39] Wauquier A, Ashton D, Marrannes R (1985). The effects of flunarizine in experimental models related to the pathogenesis of migraine. Cephalalgia.

[40] Somjen GG (2001). Mechanisms of spreading depression and hypoxic spreading depression-like depolarization. Physiol Rev.

[41] Wadman WJ, Juta AJA, kamphuis W, Somjen GG (1992). Current source density of sustained potential shifts associated with electrographic seizures and with spreading depression in rat hippocampus. Brain Res.

[42] Jing J, Aitken PG, Somjen GG (1993). Role of calcium channels in spreading depression in rat hippocampal slices. Brain Res.

[43] Leone M, Grazzi L, La Mantia L, Bussone G (1991). Flunuaizine in migraine: a minireview. Headache.

[44] Ever S, Afra J, Frese A, Goadsby PJ, Linde M, May A, Sándor PS, Members of the task force (2006). EFNS guideline on the drug treatment of migraine-report of an EFNS task force. Eur J Neurol.

[45] Richter F, Ebersberger A, Schaible HG (2002). Blockade of voltage-gated calcium channels in rat inhibits repetitive cortical spreading depression. Neurosci Lett.

[46] Shimazawa M, Hara H, Watano T, Sukamoto T (1995). Effects of Ca2+ channel blockers on cortical hypoperfusion and expression of c-Fos-like immunoreactivity after cortical spreading depression in rats. Br J Pharmacol.

[47] Nedergaard M, Hansen AJ (1988). Spreading depression is not associated with neuronal injury in the normal brain. Brain Res.

[48] Pomper JK, Haack S, Petzold GC, Buchheim K, Gabriel S, Hoffmann U, Heinemann U (2006). Repetitive normoxic spreading depression like events result in cell damage in juvenile hippocampal slice cultures. J Neurophysiol.

[49] Mies G, Paschen W (1984). Regional changes of blood flow, glucose, and ATP content determined on brain sections during a single passage of spreading depression in rat brain cortex. Exp Neurol.

[50] Hashimoto M, Takeda Y, Sato T, Kawahara H, Nagano O, Hirakawa M (2000). Dynamic changes of NADH fluorescence images and NADH content during spreading depression in the cerebral cortex of gerbils. Brain Res.

[51] Vergun O, Vltyakova TV, Reynolds IJ (2003). Spontaneous changes in mitochondrial membrane potential in single isolated brain mitochondria. Biophys J.

[52] Sangiorgi s, Mochi M, Riva R, Gortelli P, Monari L, Pierangeli G, Montagna P (1994). Abnormal platelet mitochondrial function in patients affected by migraine with and without aura. Cephalalgia.

[53] Magis D, Ambrosini A, Sándor P, Jacquy J, Laloux P, Schoenen J (2007). A randomized double-blind placebo-controlled trial of thioctic acid in migraine prophylaxis. Headache.

